# Supplementary material for: Forecasting shipping index using CEEMD-PSO-BiLSTM model
Source: PLoS One. 2023 Feb 2;18(2):e0280504. doi: 10.1371/journal.pone.0280504 (PMC9894482; doi:10.1371/journal.pone.0280504)
Supplement: Attachment — Submitted filename: Response to Reviewers.docx [file pone.0280504.s002.docx]

Dear Editor:

Thank you for the time taken to go through our manuscript! With the help of the reviewer’ comments and suggestions, substantial revisions have been made to the manuscript. Indeed, these comments and suggestions have helped to improve our paper. Please find our detailed responses below.

1. The first reviewer's comments are as follows:

The article presenting a Forecasting shipping index using CEEMD and BiLSTM model. I recommend to proceed this article to publication as it meet the requirements:1. The manuscript presented in an intelligible fashion and written in standard English.2. The authors made all data underlying the findings in their manuscript fully available.3. The manuscript technically sound, and do the data support the conclusions.

Reply: Thank you for taking time out of your busy schedule to review this article, and thank you for affirming this article.

2. The second reviewer's comments are as follows:

Basically, indexing and planning are key problem in producing and manufacturing. The paper investigated a significant topic which potential impacts. However, the method has not been proposed clearly. In particular, the motivation is weak, why the author use the proposed model structure rather than other existing models. The benefits of the proposed algorithm has not been fully reflected. The result shows the performance of the method however the comparative study is not sufficient. More comparison to the intelligent optimisation methods such as GA, PSO, should be discussed in the revised version.

Reply: Thank you for taking time out of your busy schedule to make suggestions for this article. According to your suggestion, we have done the following:

(1) Based on the Reviewer's opinion that we lack optimization algorithm, we have learned and added an improved PSO model as the optimization model of this paper. PSO has shown its powerful optimization ability in many fields, and we have used PSO in this model to produce good results. We added sections 2.4 and 3.5 to show how we optimized the CEEMD-BiLSTM model with PSO.

(2) We apologize for the lack of motivation in previous editions of the manuscript. We have added lines 70-84 in the Introduction to illustrate the purpose and innovation points of this paper. and sincerely hope that the motivation in our new edition will be stronger.

Lines 70 to 84 are as follows:

This article adopts the idea of "decomposition, reconstruction and integration" to construct a comprehensive model -- CEEMD-PSO-BiLSTM model, aiming to analyze and predict the internal characteristics and trend of shipping index, grasp the dynamic trend of shipping market, and prevent the major risks that may be brought by shipping market. At the same time, the prediction effect of deep learning in shipping index is explored to fill the gap of neural network in the field of shipping index prediction. The contribution of this article are as follows: (1) We introduce the neural network algorithm system into the shipping market prediction, and through the current better CEEMD method, the original sequence is decomposed, which creates favorable conditions for accurately fitting the nonlinear and high noise characteristics of shipping index, and significantly reduces the difficulty of neural network prediction. (2) We choose BiLSTM model with strong generalization ability in deep learning models as the framework, and combine BiLSTM model with CEEMD model in the field of signal decomposition to build a high-precision combined prediction model based on shipping index, providing a practical and reliable modeling scheme. (3) PSO was introduced to optimize the BiLSTM model, which significantly improved the prediction accuracy of shipping index.

（3）We have revised the title and conclusion of this paper. The original title is "Forecasting shipping index using CEEMD and BiLSTM model". The revised title is "Forecasting shipping index using CEEMD-PSO-BiLSTM model". In the conclusion of this paper, we add the prediction effect of CEEMD-PSO-BiLSTM model in six shipping indexes. And updated the conclusion, we think CEEMD-PSO-BiLSTM model has better prediction effect.

Thank you again for your careful review of the article, and we would appreciate any suggestions.

Sincerely yours
